# Congenital Sensorineural Deafness in Dalmatian Dogs Associated with Quantitative Trait Loci

**DOI:** 10.1371/journal.pone.0080642

**Published:** 2013-12-04

**Authors:** Susanne Kluth, Ottmar Distl

**Affiliations:** Institute for Animal Breeding and Genetics, University of Veterinary Medicine Hannover, Hannover, Germany; University of Auckland, New Zealand

## Abstract

A genome-wide association study (GWAS) was performed for 235 Dalmatian dogs using the canine Illumina high density bead chip to identify quantitative trait loci (QTL) associated with canine congenital sensorineural deafness (CCSD). Data analysis was performed for all Dalmatian dogs and in addition, separately for brown-eyed and blue-eyed dogs because of the significant influence of eye colour on CCSD in Dalmatian dogs. Mixed linear model analysis (MLM) revealed seven QTL with experiment-wide significant associations (-log_10_P>5.0) for CCSD in all Dalmatian dogs. Six QTL with experiment-wide significant associations for CCSD were found in brown-eyed Dalmatian dogs and in blue-eyed Dalmatian dogs, four experiment-wide significant QTL were detected. The experiment-wide CCSD-associated SNPs explained 82% of the phenotypic variance of CCSD. Five CCSD-loci on dog chromosomes (CFA) 6, 14, 27, 29 and 31 were in close vicinity of genes shown as causative for hearing loss in human and/or mouse.

## Introduction

Canine congenital sensorineural deafness (CCSD) is the most common cause of deafness in dogs and has been identified in more than 90 dog breeds with highest prevalences in Dalmatian dogs, English setters, English cocker spaniels, bull terriers, Australian cattle dogs, Whippets, Catahoula leopard dogs, border collies and Jack Russell terriers [1]–[3]. Dalmatian dogs are known to be afflicted from CCSD more often than other dog breeds [1]–[10]. An association between phenotypic traits and sensorineural hearing loss had been proven as the occurrence of at least one blue eye leads to a significantly higher deafness prevalence and therefore is a prominent risk factor to CCSD [2]–[3], [5]–[9]. The risk to be affected by CCSD is 2.7-times higher for blue-eyed Dalmatian puppies than in brown-eyed Dalmatian puppies [8]. In addition, presence of blue eye colour showed a genetic correlation with CCSD of 0.53 indicating that genes causing the lack of melanocytes in the eyes may play a role for CCSD [8]. Histological findings classified CCSD as Scheibe dyplasia or cochleo-saccular degeneration characterized by an initial destruction of the stria vascularis, the lateral wall of the scala media of the cochlea. The strial degeneration is followed by a degeneration of the organ of Corti, a collapse of Reissner's membrane and eventually a collapse of the whole cochlear duct. The reason for the stria vascularis degeneration has not yet been completely clarified, but absence of intermediate cells (melanocytes) is assumed to be a main factor [1], [11]–[15]. Melanocytes are the only cell type in the stria vascularis that express the potassium channel protein KCNJ10. Elimination of Kcnj10 in knockout-mice reduced endolymph potassium levels and may be the direct cause of deafness [16].

In addition, primary degenerative defects of the tectorial membrane and of the inner ear hair cells were identified to lead to CCSD in Dalmatian dogs [11], [13]. Inheritance of CCSD in Dalmatian dogs appears to be complex with heritabilities at h^2^ = 0.20–0.34 [7]–[10]. A complex segregation analysis revealed a mixed monogenic-polygenic inheritance with a major recessive gene as the best model to explain inheritance of CCSD after introducing eye colour as covariate [9]. Genes causative for CCSD in dogs have not yet been identified [1]–[3]. Previous studies found mutations in genes on dog chromosomes (CFA) 10 (*SILV, premelanosome protein*http://www.genenames.org/data/hgnc_data.php?hgnc_id=11190), 15 (*KITLG*, *KIT ligand*) and 20 (*MITF*, *microphthalmia–associated transcription factor*) associated with pigmentation patterns. However, none of these mutations provided an explanation for the occurrence of CCSD [1], [3], [17]–[21]. Neither in *SOX10* (*sex determining region Y)-box 10*, located on CFA10), which is one of the genes involved in Waardenburg syndrome [1] and a candidate gene for CCSD in Australian stumpy-tail cattle dogs [19] nor in *KITLG* which is linked to the Irish spotting phenotype, were found mutations explaining CCSD [1]. Approximately 3.5-kb upstream of the M promoter of *MITF*, two mutations associated with pigmentation patterns were determined [20]. Within the 100 kb region containing *MITF*, a microsatellite marker has been reported to show a weak association with CCSD in Dalmatian dogs [21].

Much progress has been made in identifying genes causal for human deafness related to syndromic or non-syndromic hearing impairment [22]–[29], but little is known about the complex forms of hearing loss and their genetics (The Hereditary Hearing Loss Homepage: http://webhost.ua.ac.be/hhh/). Currently 135 loci and 55 genes for monogenic forms of human deafness have been identified.

The objective of the present genome-wide association study (GWAS) was to identify loci associated with CCSD in Dalmatian dogs employing the canine high density Illumina bead chip (Illumina, San Diego, CA, USA). Within these loci and their vicinity, we searched for functional candidate genes that play a role in human deafness and genes that are involved in the development of the inner ear as well as in the differentiation and migration of melanoblasts.

## Results

**Figure 1:**
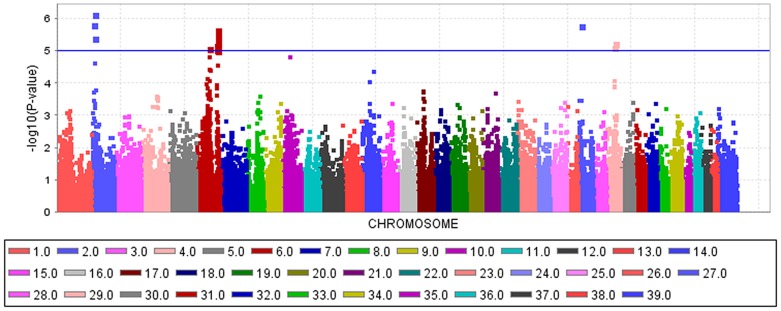
Manhattan plot of the genome-wide association study for canine congenital sensorineural deafness (CCSD) in Dalmatian dogs with brown eye colour using a mixed linear model analysis. The genome-wide -log_10_P-values for each SNP effect are plotted against its position on each chromosome. Chromosomes are differentiated by colours and numeration. Colours for chromosomes are given below the plot. The blue line indicates the significance threshold for experiment-wide significance at P<0.05.

**Figure 2:**
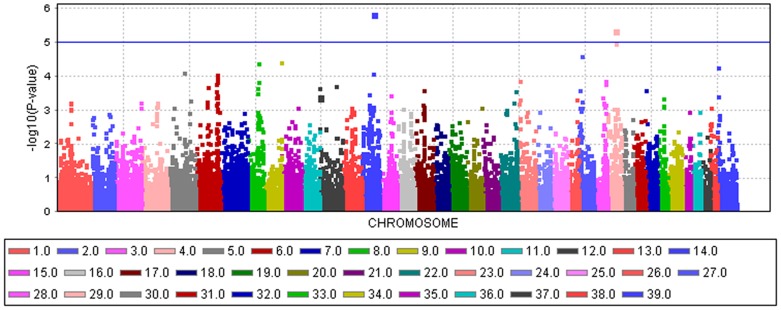
Manhattan plot of the genome-wide association study for canine congenital sensorineural deafness (CCSD) in bilaterally deaf Dalmatian dogs with brown eye colour using a mixed linear model analysis. The genome-wide -log_10_P-values for each SNP effect are plotted against its position on each chromosome. Chromosomes are differentiated by colours and numeration. Colours for chromosomes are given below the plot. The blue line indicates the significance threshold for experiment-wide significance at P<0.05.

**Figure 3:**
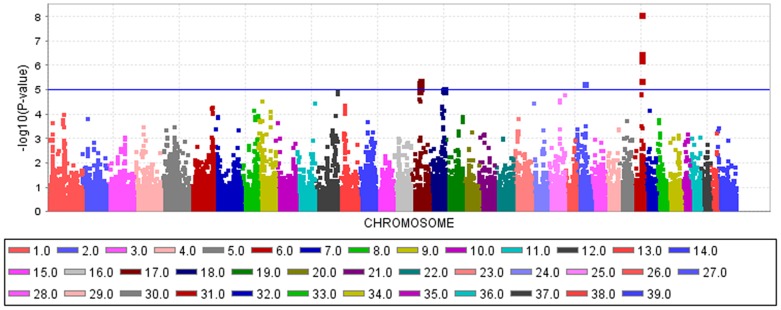
Manhattan plot of the genome-wide association study for canine congenital sensorineural deafness (CCSD) in Dalmatian dogs with blue eye colour using a mixed linear model analysis. The genome-wide -log_10_P-values for each SNP effect are plotted against its position on each chromosome. Chromosomes are differentiated by colours and numeration. Colours for chromosomes are given below the plot. The blue line indicates the significance threshold for experiment-wide significance at P<0.05.

An mixed linear model (MLM) analysis across all Dalmatian dogs revealed seven loci on dog chromosomes (CFA) 2, 6 (68 Mb), 14, 17, 27 (9 Mb), 29 and 31 as experiment-wide significant as well as three loci on CFA6 (45 Mb), 18 and 27 (25 Mb) did reach –log_10_P-values >4.0 (Table S1). The GWAS using a MLM analysis for brown-eyed Dalmatian dogs discovered five loci on dog chromosomes (CFA) 2, 6 (two loci), 27 and 29 for CCSD (Figure 1). In addition, the QTL on CFA14 was experiment-wide significantly associated with CCSD in Dalmatian dogs with brown eye colour when the dependent variate accounted for unilaterally and bilaterally affected dogs (Figure 2). These six loci had been also identified using the MLM without accounting for eye color. Four CCSD-loci were detected on CFA17, 18, 27 and 31 for blue-eyed Dalmatian dogs employing a MLM analysis (Figure 3). These four loci detected by the MLM analysis stratified for blue eye colour were also consistent with the loci found in the MLM analysis across all Dalmatian dogs. A summary of CCSD-associated loci is shown in Table S2. All –log_10_P-values of the MLM analyses were corrected for multiple testing using the adaptive false discovery rate according to Benjamini-Hochberg. The thresholds for experiment-wide significance at P-values <0.05, 0.01 and 0.001 were at observed –log_10_P-values >5.0, 6.3 and 8.0. The observed -log_10_P-values were plotted against the expected -log_10_P-values and the quantile-quantile plots indicated that the population stratification was eliminated through the identity-by-state (IBS) kinship matrix and the fixed effect of sex (Figures S1–S3). On CFA2, there were two experiment-wide significantly CCSD-associated SNPs within an interval of 19.778 kb and a further significant CCSD-SNP at 13.139 Mb of the CanFam3.1 genome assembly. On CFA6 at 69 Mb, there were 19 experiment-wide significantly CCSD-associated SNPs within an interval of 1.287 Mb. On CFA17, eight SNPs within an interval of 1.830 Mb, on CFA18 six SNPs within an interval of 1.012 Mb, on CFA27 two SNPs within an interval of 0.018 Mb and on CFA31 seven SNPs within an interval of 0.743 Mb were experiment-wide significantly associated with CCSD.

In Table 1, from each QTL the SNP with the highest –log_10_P-value from the MLM analysis is given. The odds ratios (ORs) stratified by sex were determined to demonstrate the impact of the significantly associated SNPs on CCSD. The ORs for CCSD in Dalmatian dogs with brown eyes were at 1.18–10.70 and for blue-eyed Dalmatian dogs at 1.73–7.85. The single SNPs with the highest –log_10_P-values from each QTL explained between 11 and 14% of the phenotypic variance for CCSD in brown-eyed Dalmatian dogs and the respective SNPs for blue-eyed Dalmatian dogs between 15 and 26%. The SNP BICF2P176848 on CFA2 explained the largest proportion of variance for brown-eyed Dalmatian dogs and for blue-eyed Dalmatian dogs, the SNP BICF2G630740465 on CFA31. The variance explained by all experiment-wide significant SNPs for brown-eyed Dalmatian dogs was at 65.5% in a general linear model, for blue-eyed Dalmatian dogs at 64.4% and for all Dalmatian dogs at 82.4%.

**Table 1:** Summary of results for the genome-wide association study using a mixed linear model and a sex-stratified case-control analysis for canine congenital sensorineural deafness in Dalmatian dogs.

Trait	Position	Position	SNP-ID	Minor allele	MAF	MAF_a_	MAF_u_	VE	OR	CI-L	CI-U	-log_10_P	Candidate gene	Distance to the nearest candidate gene in Mb
CFA	CanFam3.1	CanFam2.0												
Deafness in brown eyed dogs														
2	13,786,700	16,675,682	BICF2P176848	T	0.08	0.19	0.03	0.14	7.76	3.48	17.30	6.15	*ARHGAP12*	1.19 (-)
6	45,474,835	48,537,551	TIGRP2P83893_RS8732055	A	0.49	0.69	0.42	0.12	3.13	1.96	5.00	5.09	*COL11A1*	1.95 (−)
6	68,927,940	71,986,567	BICF2P590845	A	0.14	0.15	0.13	0.13	1.30	0.71	2.33	5.68	*GIPC2*	0.06 (+)
14	39,561,348	42,518,712	BICF2G630529431	T	0.17	0.13	0.18	0.13	1.41	0.77	2.56	5.85	*DFNA5*	1.29 (+)
													*HOXA1*	0.71 (−)
27	9,400,352	12,412,335	BICF2S23410492	C	0.05	0.17	0.02	0.11	10.70	3.87	29.6	5.80	*TWF1*	0.88 (−)
29	23,903,462	26,901,967	BICF2G630625485	T	0.20	0.23	0.20	0.12	1.18	0.72	1.96	5.26	*GDAP1*	1.07 (+)
Deafness in blue eyed dogs														
17	28,929,911	32,032,913	BICF2G630212376	A	0.11	0.17	0.08	0.17	2.20	0.78	6.25	5.41	*CRIM1*	intragenic
18	51,795,260	54,820,227	BICF2P28982	G	0.16	0.24	0.15	0.15	1.73	0.75	4.01	5.08	*CDC42EP2*	0.06 (−)
27	25,549,421	28,568,843	BICF2P507470	T	0.11	0.35	0.07	0.15	7.85	3.40	18.1	5.32	*AEBP2*	2.11 (−)
31	30,836,962	32,955,387	BICF2G630740465	G	0.20	0.44	0.16	0.26	4.18	1.94	8.98	8.13	*CLDN14*	0.84 (−)

The SNP-ID, the position on dog chromosome (CFA) in base pairs (bp) according to CanFam3.1 and CanFam2.0, minor allele, minor allele frequency (MAF) for all, affected (MAF_a_) and unaffected (MAF_u_) dogs (controls), variance explained by the respective SNP (VE) and -log_10_P-values (-log_10_P) from the mixed linear model analysis and positional candidate genes with their distance to the CCSD-associated SNP are given. SNPs downstream of the nearest candidate gene are marked with a plus sign (+) and SNPs upstream of the nearest candidate gene are marked with a minus sign (−). Odds ratios (OR) are from a case-control study stratified by sex with 95% confidence intervals (CI).

Haplotype structure and haplotype association were analyzed for all ten genomic regions surrounding the significantly associated SNPs (Figures 4, 5, 6, 7, 8, 9, 10, 11, 12, 13). The SNPs BICF2P176848 on CFA2, TIGRP2P83893_RS8732055 on CFA6 and BICF2P507470 on CFA27 reside within a significantly (P-value<0.05 after 10,000 permutations) CCSD-associated haplotype block, respectively (Figures 4,5,12).

**Figure 4:**
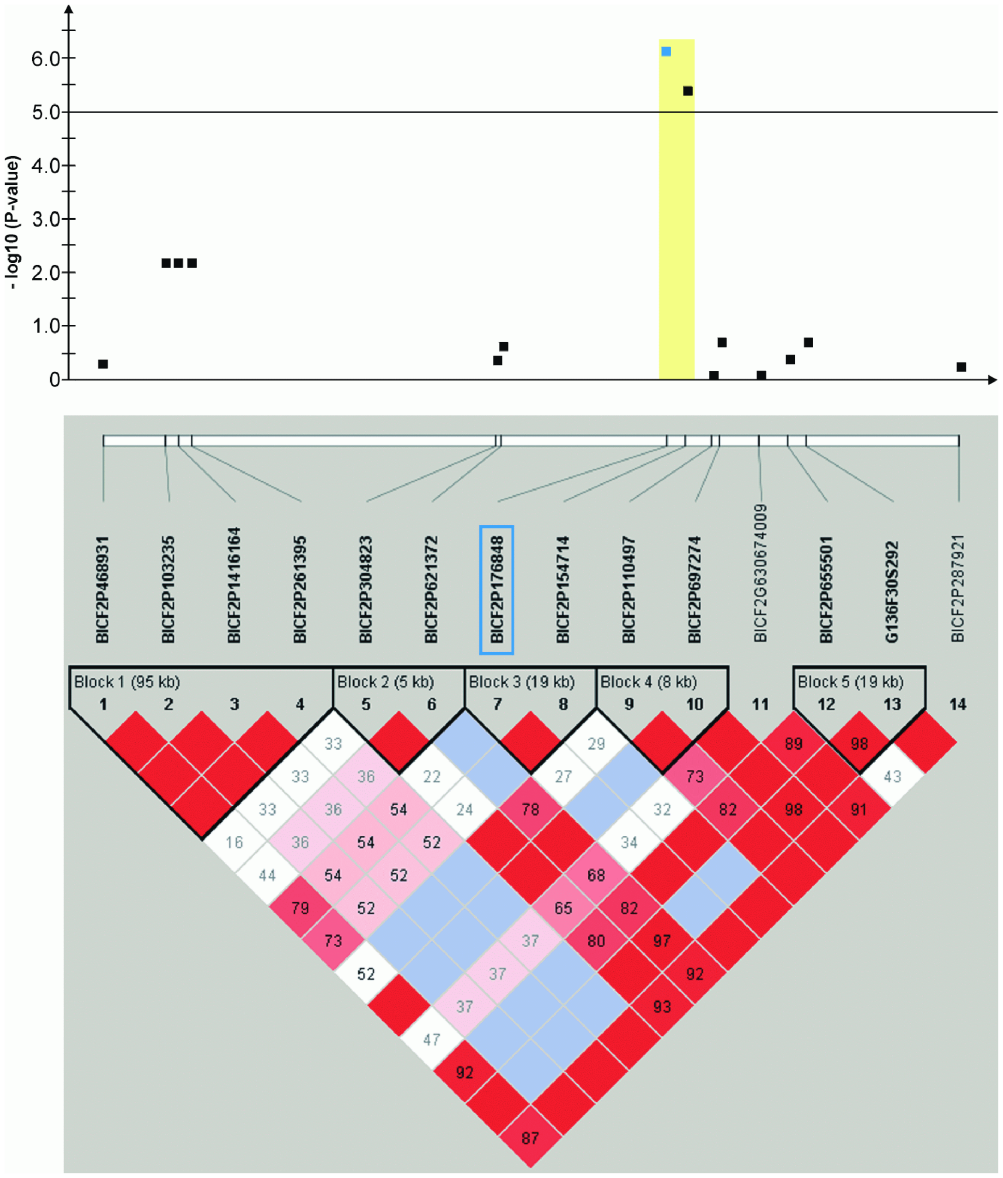
Genome-wide association for congenital sensorineural deafness in brown-eyed Dalmatian dogs on dog chromosome (CFA) 2. The –log_10_P-values of all 14 SNPs with their chromosomal positions on CFA2 and their haplotype structure are shown at 13.1–14.1 Mb. Significantly (P-value<0.05) CCSD-associated haplotype blocks are blocks 1, 3 and 5. Block 3 (13.78–13.80 Mb) harbours the experiment-wide significantly associated SNP BICF2P176848.

**Figure 5:**
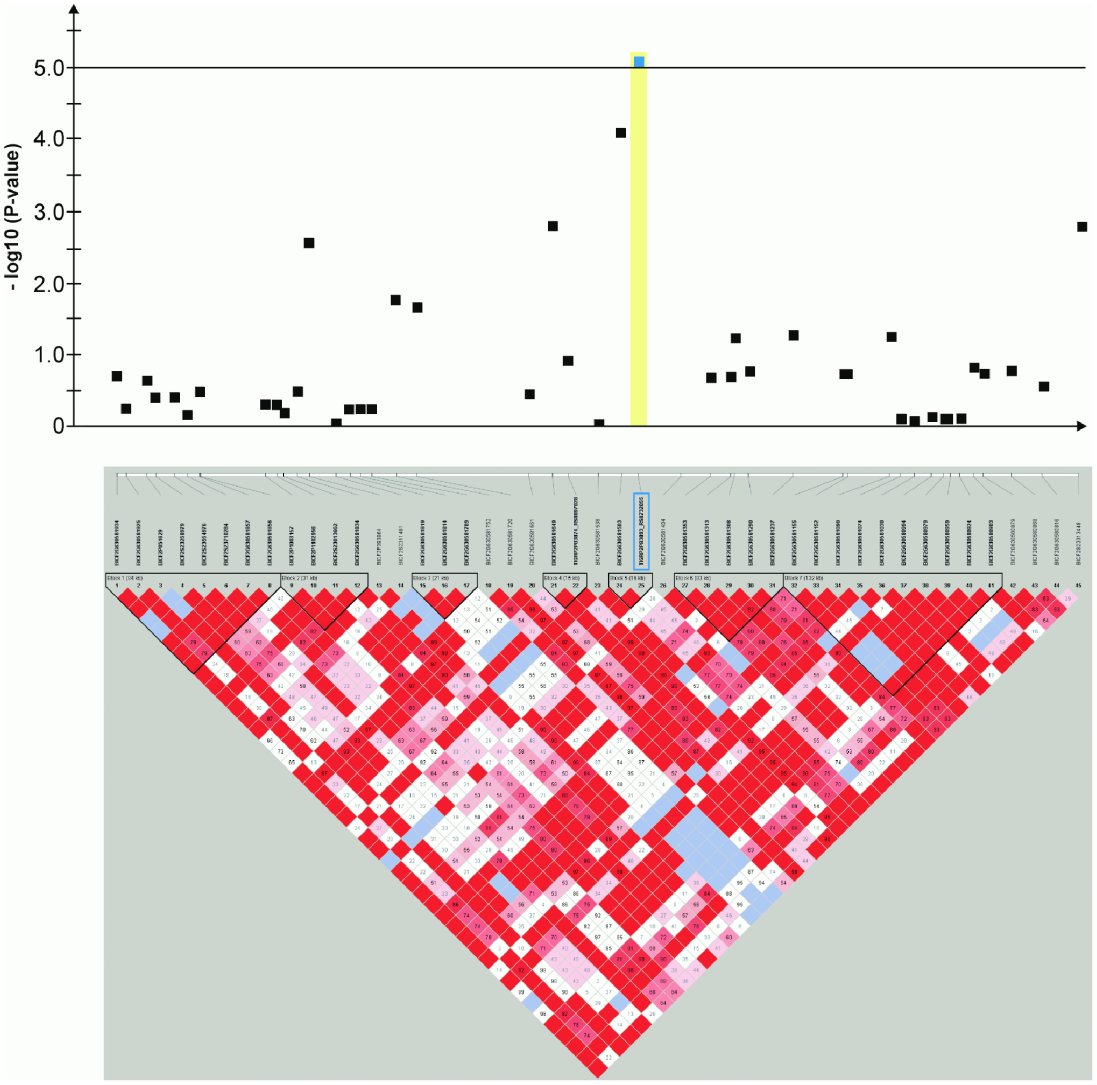
Genome-wide association for congenital sensorineural deafness in brown-eyed Dalmatian dogs on dog chromosome (CFA) 6. The –log_10_P-values of all 45 SNPs with their chromosomal positions on CFA6 and their haplotype structure are shown at 44.9–45.9 Mb. Significantly (P-value<0.05) CCSD-associated haplotype blocks are blocks 1, 4, 5, 6 and 7. Block 5 (45.45–45.47 Mb) harbours the experiment-wide significantly associated SNP TIGRP2P83893_RS8732055.

**Figure 6:**
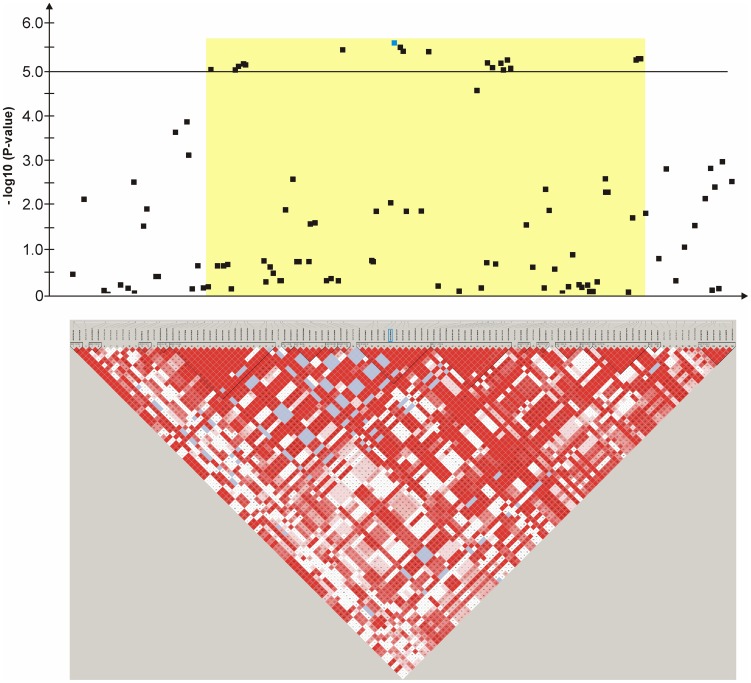
Genome-wide association for congenital sensorineural deafness in brown-eyed Dalmatian dogs on dog chromosome (CFA) 6. The –log_10_P-values of all 107 SNPs with their chromosomal positions on CFA6 and their haplotype structure are shown at 67.9–69.9 Mb. Significantly (P-value<0.05) CCSD-associated haplotype blocks are blocks 8, 12, 16 and 18. The SNP BICF2S23746914 is located within intron 6 of *GIPC2*.

**Figure 7:**
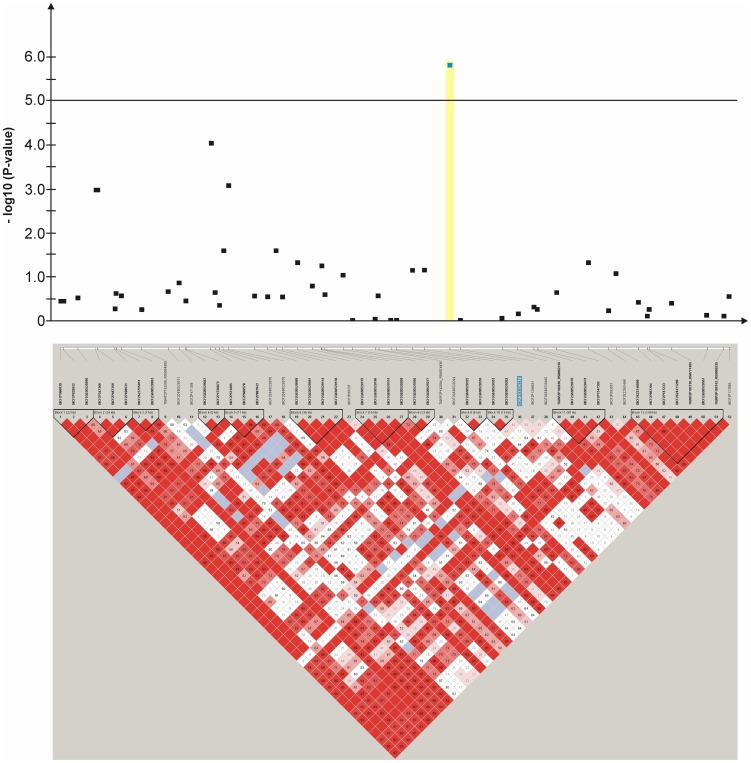
Genome-wide association study for congenital sensorineural deafness in brown-eyed Dalmatian dogs on dog chromosome (CFA) 14. The –log_10_P-values of all 52 SNPs with their chromosomal positions on CFA14 and their haplotype structure are shown at 39.0–39.9 Mb. Significantly (P-value<0.05) CCSD-associated haplotype blocks are blocks 2, 5, 6, 7, 11 and 12.

**Figure 8:**
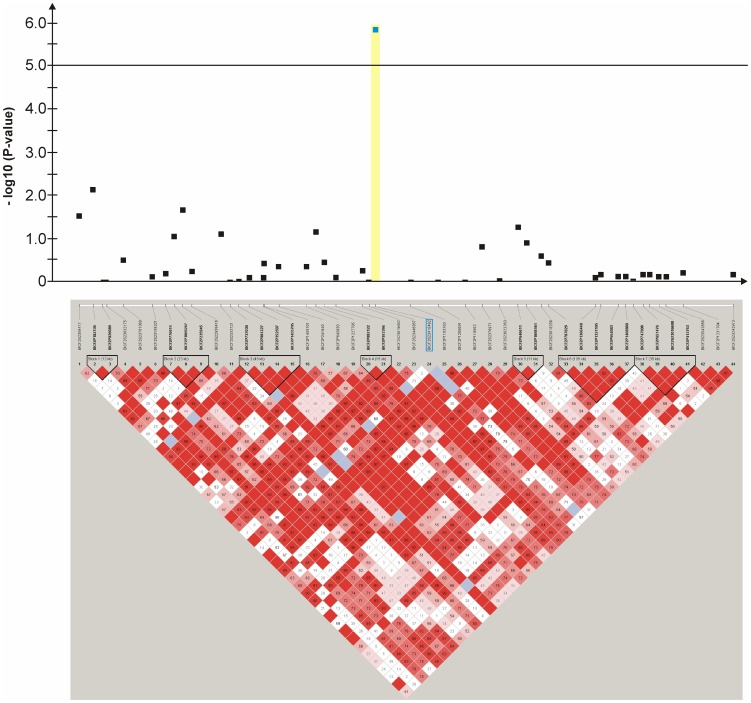
Genome-wide association study for congenital sensorineural deafness in brown-eyed Dalmatian dogs on dog chromosome (CFA) 27. The –log_10_P-values of all 44 SNPs with their chromosomal positions on CFA27 and their haplotype structure are shown at 8.9–9.8 Mb. Significantly (P-value<0.05) CCSD-associated haplotype blocks are blocks 1, 4 and 6.

**Figure 9:**
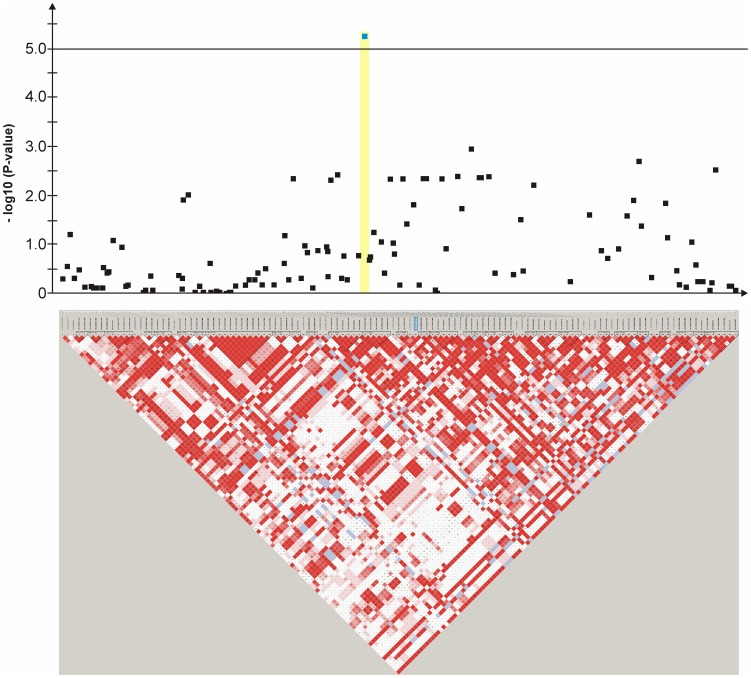
Genome-wide association study for congenital sensorineural deafness in brown-eyed Dalmatian dogs on dog chromosome (CFA) 29. The –log_10_P-values of all 121 SNPs with their chromosomal positions on CFA29 and their haplotype structure are shown at 23.0–24.9 Mb.

**Figure 10:**
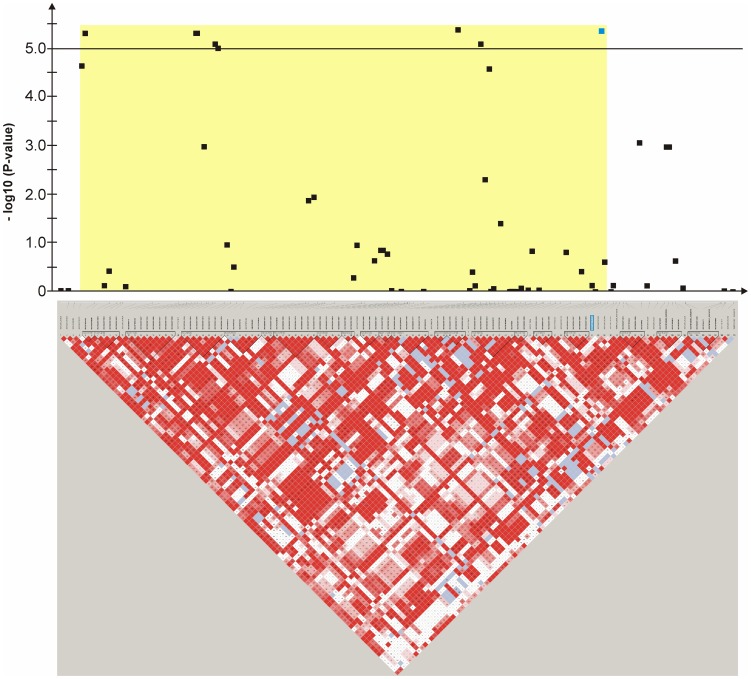
Genome-wide association study for congenital sensorineural deafness in blue-eyed Dalmatian dogs on dog chromosome (CFA) 17. The –log_10_P-values of all 105 SNPs with their chromosomal positions on CFA17 and their haplotype structure are shown at 26.9–29.3 Mb. The experiment-wide significantly associated SNP BICF2G630212376 is located within intron 2 of *CRIM1*.

**Figure 11:**
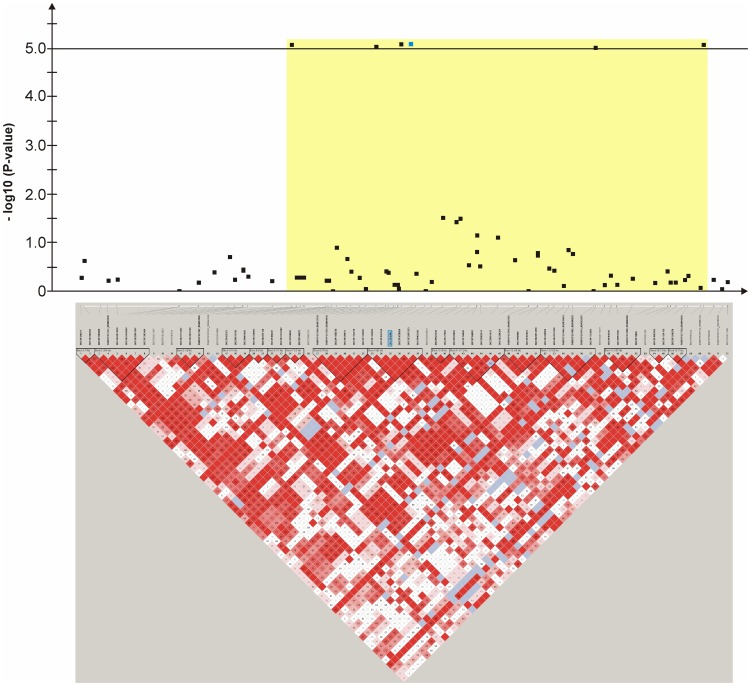
Genome-wide association study for congenital sensorineural deafness in blue-eyed Dalmatian dogs on dog chromosome (CFA) 18. The –log_10_P-values of all 72 SNPs with their chromosomal positions on CFA18 and their haplotype structure are shown at 50.9–52.5 Mb.

**Figure 12:**
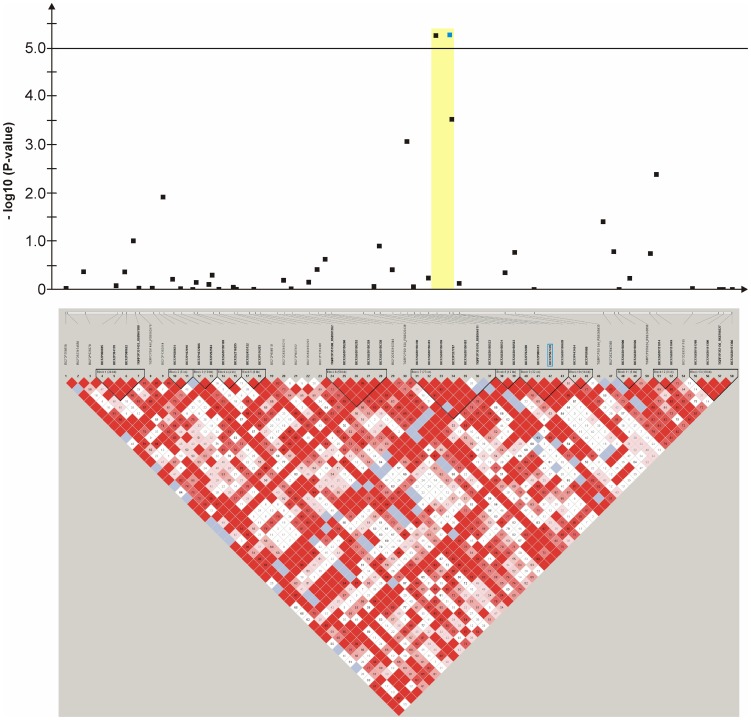
Genome-wide association study for congenital sensorineural deafness in blue-eyed Dalmatian dogs on dog chromosome (CFA) 27. The –log_10_P-values of all 57 SNPs with their chromosomal positions on CFA27 and their haplotype structure are shown at 24.9–25.9 Mb. Significantly (P-value<0.05) CCSD-associated haplotype blocks are blocks 6, 7, 9 and 12. Block 9 (25.52–25.55 Mb) harbours the experiment-wide significantly associated SNP BICF2P507470.

**Figure 13:**
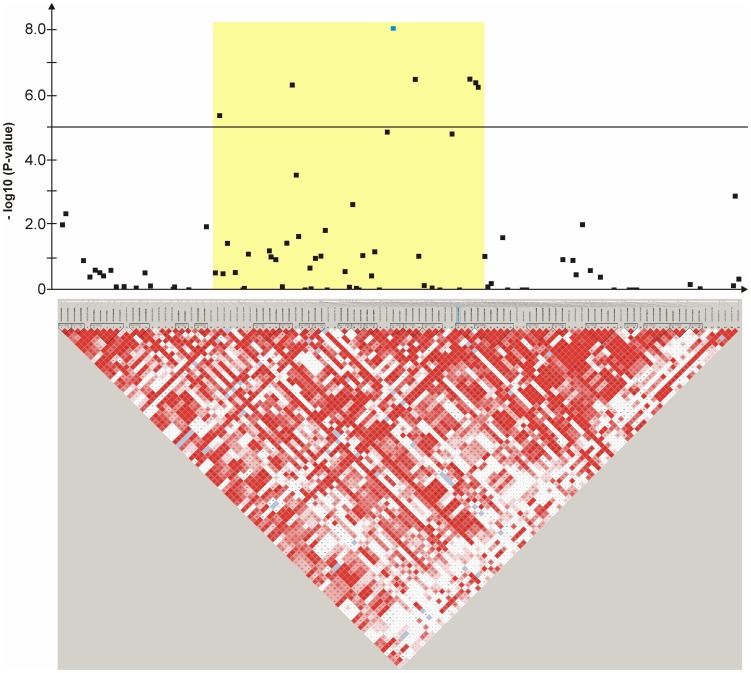
Genome-wide association for congenital sensorineural deafness in blue-eyed Dalmatian dogs on dog chromosome (CFA) 31. The –log_10_P-values of all 105 SNPs with their chromosomal positions on CFA31 and their haplotype structure are shown at 29.8–31.8 Mb.

**Figure 14:**
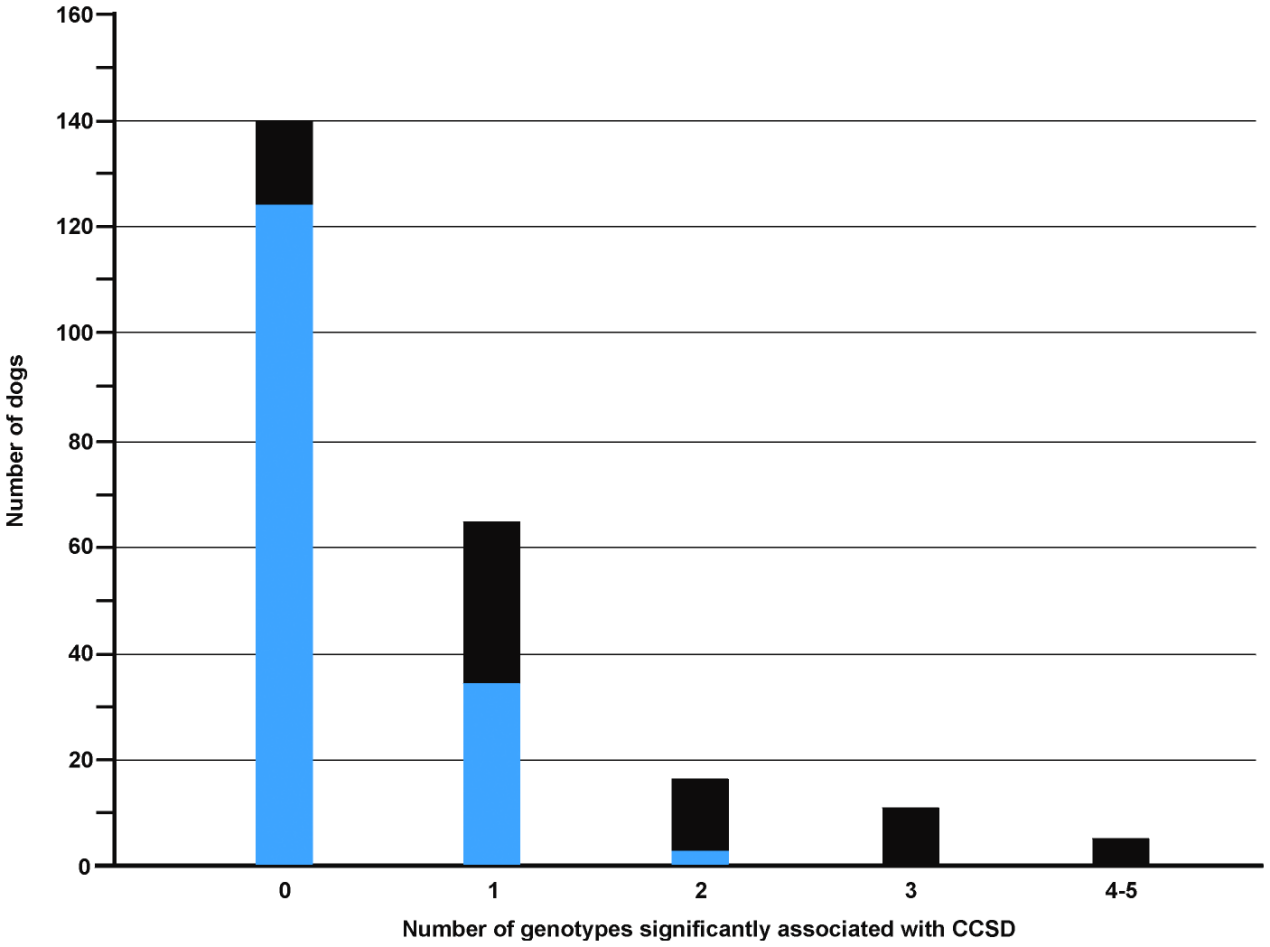
Cumulative distribution of the number of genotypes per individual dog associated with canine congenital sensorineural deafness (CCSD) by unaffected and CCSD-affected Dalmatian dogs. From each QTL, only the highest CCSD-associated genotype had been used. The section of the bars with blue colour represents the number of unaffected and the section of the bars with the black colour CCSD-affected Dalmatian dogs.

The cumulative distribution of CCSD-associated genotypes per individual dog among affected and unaffected animals shows that at least one CCSD-predisposing genotype significantly increases the risk to CCSD (Figure 14). In the case that Dalmatian dogs carry more than one CCSD-associated genotype, nearly all of them are affected. The distribution of the SNP-genotypes also shows that animals with susceptible genotypes are at high risk to CCSD (Table S3 and S4).

## Discussion

The present GWAS for CCSD in Dalmatian dogs identified seven experiment-wide significant CCSD-loci for Dalmatian dogs, whereof six were experiment-wide significant for brown-eyed Dalmatian dogs and four experiment-wide significant for blue-eyed Dalmatian dogs. The QTL among brown-eyed and blue-eyed Dalmatian dogs were located on different chromosomal positions and appeared largely independently associated with CCSD. The presence of at least one CCSD-predisposing genotype significantly increased the risk to CCSD. Therefore, we assume that several loci contribute to the complex inheritance of CCSD in Dalmatian dogs [7]–[10]. Our study is the first GWAS for CCSD in Dalmatian dogs and we can demonstrate that a large proportion of the phenotypic variation of this trait can be captured through the significantly associated SNPs.

The models we employed for the GWAS should fully account for the strong stratification of iris pigmentation on the prevalence of CCSD. Therefore, the association analyses were separately performed for brown-eyed and blue-eyed Dalmatian dogs. This type of parameterization allowed us to infer associations in dependence of eye pigmentation genes and to differentiate among loci causing genetic heterogeneity. An analysis across all Dalmatian dogs without taking into account the stratification caused by iris pigmentation may mask some loci or underestimate associations due to genetic heterogeneity. We employed an MLM analysis across all Dalmatian dogs without accounting for iris pigmentation in order to see which loci might be affected by this stratification effect. This analysis revealed the loci on CFA2, 6 (68 Mb), 14, 17, 27 (9 Mb), 29 and 31 as experiment-wide significant, whereas the loci on CFA6 (45 Mb), 18 and 27 (25 Mb) did not reach experiment-wide significance thresholds. Possibly, these latter loci on CFA6, 18 and 27 may be more strongly influenced by a segregation pattern which is different among Dalmatian dogs with different iris colour due to genetic heterogeneity. Accounting for eye colour may also facilitate replication studies as CCSD-predisposing alleles of some loci seem to be more common in blue-eyed versus brown-eyed Dalmatian dogs.

Hearing loss is unilateral or bilateral in Dalmatian dogs [1]–[10]. We observed a random segregation of the unilateral and bilateral CCSD-affection status in closely related dogs and thus a genetic correlation close to one. In addition, the GWAS showed very similar results treating all CCSD-affected individuals as cases or differentiating among unilaterally and bilaterally CCSD-affected cases. The distribution of CCSD-associated genotypes per individual dog suggested that dogs with bilateral hearing loss had CCSD-susceptible genotypes at several loci and therefore, accumulated a larger number of CCSD-predisposing alleles from several loci than dogs with unilateral hearing loss. These loci appeared randomly distributed without a clear pattern for a preference of specific loci. An unequal distribution of CCSD-associated alleles may be due to Mendelian segregation among full-sibs.

In agreement with previous studies we did not find experiment-wide significant associations with markers located within *MITF*, *SILV*, *SOX10* and *KITLG* or in the neighbourhood of *MITF*, *SILV* and *KITLG* [17]–[21]. A marker in the vicinity of *MITF* showed an association at a nominal P-value<0.05 in German Dalmatian dogs but did not exceed the experiment-wide significance level [21]. This association could also be confirmed in the present data set (data not shown). The linkage interval on CFA10 in the Australian stumpy-tail cattle dog covered most of the chromosome and thus, *SOX10* may be one of many other candidates [19].

Candidate genes were identified using the dog genome assembly build 3.1 [http://www.ensembl.org/Canis_familiaris/]. In the neighbourhood of four experiment-wide significant CCSD-loci, genes were found that are known to be involved in human hearing loss including *COL11A1* (*collagen type XI alpha 1*) on CFA6, *DFNA5* and *HOXA1* (*homeobox A1*) on CFA14, *GDAP1* (*ganglioside induced differentiation associated protein 1*) on CFA29 and *CLDN14* (*claudin-14*) on CFA31. These genes are involved in the development and maintenance of inner ear structures [22]–[29].

Four CCSD-loci were close to genes influencing the migration of melanocytes and the melanosome transport [30]–[34]. An implication of these possible candidate genes for CCSD has not yet established but should drive further research. The role of genes influencing melanocyte differentiation and migration is worth further investigation as these genes may affect pathways of genes involved in Waardenburg syndrome (WS) [28]. Those genes are *ARHGAP12* (*Rho GTPase activating protein 12*) on CFA2, *TWF1* (*twinfilin, actin-binding protein, homolog 1 (Drosophila)*) on CFA27, *CDC42EP2* on CFA18 and *AEBP2* (*AE (adipocyte enhancer) binding protein 2*) on CFA27.

Further two genes, *GIPC2* (*GIPC PDZ domain containing family, member 2*) on CFA6 and *CRIM1* (*cysteine rich transmembrane BMP regulator 1 (chordin-like)*) on CFA17 showed significant associations through SNPs located within these genes. *CRIM1* is associated with pleiomorphic phenotypes in mutant mice [35] and possibly with melanoblast migration [36].

*DFNA5, COL11A1* and *HOXA1* influence cochlear development and morphogenesis, and cause sensorineural hearing loss in humans when mutated. Gain of function mutations in *DFNA5* lead to pathological programed cell death in the differentiated auditory sensory epithelium of the organ of Corti [37]–[41], while dominant mutations in *COL11A1* cause the production of mutant collagen resulting in abnormally arranged collagen fibrils within the tectorial membrane [42]–[47]. Truncating mutations in *HOXA1* are associated to malformations of the inner ear [48]–[49]. The CCSD-locus on CFA14 is located in between the genes *DFNA5* and *HOXA1* and its association is due to bilaterally deaf Dalmatian dogs and thus, these dogs may be affected by mutations of both genes, *DFNA5* and *HOXA1*.

Mutations in the gene *GDAP1* are involved in the Charcot-Marie-Tooth disease (CMT) type 2 K and 4 A in humans that is characterized by progressive hypo- and demyelination resulting in motor and sensory neuropathy. Rare cases of CMT show neural deafness as a clinical feature in addition to other symptoms [50]–[51].

Within the QTL on CFA6 at 69 Mb, *GIPC2* is located. Even if this gene has no obvious known contribution to CCSD, it may possibly influence the maintenance of inner ear structures as mutations in its family members, *GIPC1* and *GIPC3*, are able to cause sensorineural hearing loss due to degeneration of hair cells and ganglion neurons of the nervus cochlearis [52]–[55].

The candidate genes *ARHGAP12* and *TWF1* on CFA2 and CFA27 possibly interact due to cytoskeletal organisation during melanocyte migration and melanosome transfer. The association of piebaldism with deafness may be due to the lack of melanoblast-derived intermediate cells of the stria vascularis because degeneration of the organ of Corti is observed in several mouse models when these cells are missing [56]. The formation of lamellipodia, filopodia and stress fibres is essential for melanocyte functions. Changes of cell shape emerge from cytoskeletal organization orchestrated by the family of GTPases, like Rac, Rho or Cdc42 [57]. ARHGAP12 is able to regulate Rho, which induces dissolution of stress fibres [58]. Furthermore, *TWF1* is regulated by the small GTPases Rac1 and Cdc42 and is involved in cell shape modification [59]. Interestingly, the family member *Twinfilin2 (TWF2)* influences the development and maintenance of sterocilia. Sterocilia are actin filled and positioned on the apical surface of hair cells required for mechanoelectrical transduction within the inner ear [29], [60]. However, cytoskeletal rearrangement orchestrated by *TWF1* might be involved in melanocyte dendricity but also hair-bundle development.

In blue-eyed Dalmatian dogs, the GWAS detected a QTL close to *CLDN14*. Mutations of *CLDN14* cause sensorineural hearing loss in human and mice. Hearing loss in these cases is due to rapid degeneration of the outer hair cells and later on, of the inner hair cells [61]–[65].

The candidate genes on CFA18 and 27 play a role in melanocyte dendricity and development. The CFA18 candidate gene *CDC42EP2* interacts with the small GTP-binding protein Cdc42. Activated Cdc42 is associated with changes in the cytoskeleton of melanocytes and is able to stimulate filopodia formation for melanocyte dendricity [57], [66]–[67].

The further candidate gene on CFA27, *Aebp2* is expressed in neural crest cells of the developing mouse embryo. Mutations in *Aebp2* in mice result in an enlarged colon, hypopigmentation and auditory defects similar to human Hirschsprung's disease and Waardenburg syndrome. Melanoblasts migrate from the neural crest through several pathways to the skin, the iris and choroids of the eye, and the inner ear. An alteration of these pathways causes the absence of melanocytes and thus depigmented areas and deafness in human and mouse [28], [29], [68]–[69].

The candidate gene *CRIM1* on CFA17 interacts with members of the transforming growth factor beta family and plays a role in tissue development of the eye of mice [35], [70]. Furthermore, a recent study identified *CRIM1* to be involved in eye area pigmentation in Fleckvieh cattle [36].

All these aforementioned candidate genes for CCSD in Dalmatian dogs share interactions mediated by co-expression as shown by a network analysis using Genemania (http://www.genemania.org).

In conclusion, the GWAS suggested that different loci may be independently associated with CCSD in Dalmatian dogs. Thus, non-allelic heterogeneity may be the underlying mode for the complex inheritance of CCSD in the Dalmatian dog. We found support for candidate genes involved in the development of the tectorial membrane, sensory epithelium of organ of Corti and ganglion neurons as well as the maintenance of cochlear hair cells. To clarify a possible involvement of these candidate genes and genes regulating cytoskeletal rearrangement and migration of melanocytes requires further research in larger samples and deep sequencing of the candidate genomic regions. The genetic approach in the Dalmatian dog may open the way to decipher the molecular mechanisms underlying CCSD and then this dog breed may be a suitable animal model for further studies in human sensorineural hearing loss.

## Materials and Methods

### Ethics Statement

All animal work was conducted according to national and international guidelines for animal welfare. The dogs of this study were included with the consent of their owners. The BAER test (brainstem auditory evoked response test) was performed in veterinary clinics. BAER testing is mandatory for all Dalmatian dogs in Germany. The blood samples were taken by veterinarians during routine examinations for the BAER test referring to good veterinary practice. Approval from the ethics committee was not obtained because blood sampling was during a diagnostic veterinary procedure according to the German Animal Welfare Law (released on 05/18/2006, last changes on 12/09/2010).

### Animals

In the present study, 235 Dalmatian dogs with a definitive diagnosis for CCSD were included (Table 2). Among these were 157 Dalmatian dogs with normal hearing and either brown or blue eye colour, and 61 brown-eyed dogs and 17 blue-eyed Dalmatian dogs with unilaterally or bilaterally hearing loss. The animals were balanced according to sex, ancestry and hearing loss. All dogs in the present study were not related up to the grandparent level. All Dalmatian dogs genotyped were screened for unilateral and bilateral hearing loss by trained veterinarians using the BAER test. The test was performed in six to eight weeks old puppies under general anaesthesia with xylazine and ketamine. Each ear was tested individually after obvious cases of conductive deafness were excluded using otoscopy. Standard ear phones and acquisition measurement conditions were used as well as same stimulus click rates with 30 clicks per second and 1000 clicks per ear. Stainless steel subdermal needle electrodes were used for recording. Clicks were presented at 80 db normal hearing level (nHL) to an individual ear. White noise, at 30 db lower than the stimulus, was delivered into the non tested ear to avoid crossover recordings from a functional contralateral ear. Conductive deafness was excluded using bone-conduction stimulation via a vibrator. Bone-conduction stimulation was employed through removing the vibration unit from the headset and placing it on the mastoid process of the tested ear [71], [72]. The waveform responses recorded within the first 10 ms consists of up to seven waves in normal hearing dogs [73]. Dogs with an essential flat line without any identifiable waveforms were diagnosed as deaf for the respective ear. Puppies with inexplicit test results were retested at an age of three to four months. All the puppies were classified as hearing, unilaterally deaf or bilaterally deaf.

**Table 2:** Number of Dalmatian dogs genotyped on the canine Illumina high density beadchip.

Trait	Male	Female	Total
Control	74	83	157
Uni- or bilateral CCSD and brown eye colour	29	32	61
Uni- or bilateral CCSD and blue eye colour	8	9	17
Only bilateral CCSD	10	12	22
Only bilateral CCSD and brown eye colour	9	8	17

Number of dogs stratified by sex, eye colour and canine congenital sensorineural deafness (CCSD). CCSD can be unilateral or bilateral. In addition to all cases of hearing loss including both, unilateral or bilateral cases, bilateral CCSD cases (n = 22) and bilateral CCSD cases with brown eye colour (n = 17) are given.

### DNA preparation and Genotyping

Genomic DNA was extracted from EDTA-blood samples through a standard ethanol fractionation with concentrated sodium chloride (6 M NaCl) and sodium dodecyl sulphate (10% SDS). Concentration of extracted DNA for SNP chip analysis was adjusted to 50 ng/µl using Nanodrop ND-1000 (Peqlab Biotechnology, Erlangen, Germany).

Genotyping for the 235 Dalmatian dogs was performed using the canine Illumina high density beadchip (Illumnia) with 173,662 SNPs. Quality control (QC) criteria were minor allele frequencies (MAF) >0.05, genotyping rate per SNP and animal >0.90 and Hardy-Weinberg equilibrium (P-value<0.000001). These QC criteria were met by 106,435 SNPs and this number of SNPs was used for all statistical analyses. Chromosomal positions of the SNPs were determined using the *canis lupus familiaris* genome assembly CanFam3.1 (http://www.ensembl.org/Canis_familiaris/) and BLASTN (http://www.ensembl.org/Multi/blastview).

### Statistical Analysis

The ALLELE procedure of SAS/Genetics, version 9.3 (SAS Institute, Cary, NC, USA), was used to calculate polymorphism information content, heterozygosity, allelic diversity, allele and genotype frequencies and χ^2^-tests for Hardy-Weinberg-Equilibrium. The genome-wide association analyses were performed using a mixed linear model (MLM) using TASSEL, version 3.0.88 [74]. Analyses were done for brown-eyed and blue-eyed dogs separately and encoding dogs with normal hearing status as controls and dogs with unilateral or bilateral hearing loss as cases. A further GWAS for brown-eyed dogs was employed encoding controls with 1, unilaterally CCSD-affected with 2 and bilaterally CCSD-affected with 3 in order to account for the number of ears with CCSD. The MLM included the fixed effects of sex, SNP-genotype and the random animal effect via the identity-by-state-kinship (IBS) matrix. The IBS matrix reflects the genomic relationship matrix among all individuals genotyped and captures the relatedness among animals as well as the cryptic family structure. The variance explained by each SNP was calculated employing the MLM. The adaptive false discovery rate according to Benjamini-Hochberg was calculated using the MULTIPLE TEST procedure of SAS, version 9.3, to determine the threshold for experiment-wide significance. The CASECONTROL procedure of SAS/Genetics, version 9.3, was used to calculate ORs with their 95% confidence intervals using sex as strata. The proportion of variance explained by all the CCSD-associated SNPs was jointly estimated using a general linear model and comparisons of the residual variances of the reference model with sex only (V_R_) to the models with sex and the respective SNP genotype effects (V_F_). The proportion of variance explained by the respective SNP genotypes was calculated as 1-(V_F_/V_R_). We analysed the haplotype structure of the identified QTL and their flanking genomic regions spanning 1–2 Mb and tested the association of haplotype blocks to CCSD with a permutation rate of 10.000 using Haploview 4.0 [75].

## Supporting Information

Figure S1 Q-Q-plot of expected –log_10_P-values versus observed–log_10_P-values from the mixed linear model analysis for congenital sensorineural deafness in Dalmatian dogs with brown eye colour. Shown are all 106,435 SNPs included in the genome-wide association analysis with the grey line corresponding to the null hypothesis of no association.
(DOC)

Figure S2 Q-Q-plot of expected –log_10_P-values versus observed–log_10_P-values from the mixed linear model analysis for congenital sensorineural deafness in Dalmatian dogs with bilateral deafness. Shown are all 106,435 SNPs included in the genome-wide association analysis with the grey line corresponding to the null hypothesis of no association.
(DOC)

Figure S3 Q-Q-plot of expected –log_10_P-values versus observed–log_10_P-values from the mixed linear model analysis for congenital sensorineural deafness in Dalmatian dogs with blue eye colour. Shown are all 106,435 SNPs included in the genome-wide association analysis with the grey line corresponding to the null hypothesis of no association.
(DOC)

Table S1 Summary of results for the genome-wide association study employing a mixed linear model for canine congenital sensorineural deafness in Dalmatian dogs using all deaf dogs as cases and all hearing dogs as controls. The SNP-ID, the position on dog chromosome (CFA) in base pairs (bp) according to CanFam3.1 and CanFam2.0, minor allele, minor allele frequency (MAF) for all, affected (MAF_a_) and unaffected (MAF_u_) dogs (controls), variance explained by the respective SNP (VE) and -log_10_P-values (-log_10_P) from the mixed linear model analysis are given. Odds ratios (OR) are from a case-control study stratified by sex with 95% confidence intervals (CI).
(DOC)

Table S2 Chromosomal loci identified as significantly associated with canine congenital sensorineural deafness, compared to a control group of 157 hearing Dalmatian dogs.
(DOCX)

Table S3 Distribution (%) of canine congenital sensorineural deafness (CCSD) in Dalmatian dogs by the genotypes of significantly associated SNPs. With exception of SNP BICF2P176848, only each one genotype per SNP is highly associated with CCSD-affection.
(DOC)

Table S4 Distribution (%) of the genotypes significantly associated with canine congenital sensorineural deafness (CCSD) for controls and CCSD-affected Dalmatian dogs. The genotypes conferring high risk to CCSD are given in bold.
(DOC)
