# Supplementary material for: Congenital Sensorineural Deafness in Dalmatian Dogs Associated with Quantitative Trait Loci
Source: PLoS One. 2013 Dec 4;8(12):e80642. doi: 10.1371/journal.pone.0080642 (PMC3851758; doi:10.1371/journal.pone.0080642)
Supplement: Figure S2 — Q-Q-plot of expected –log10P-values versus observed–log10P-values from the mixed linear model analysis for congenital sensorineural deafness in Dalmatian dogs with bilateral deafness. Shown are all 106,435 SNPs included in the genome-wide association analysis with the grey line corresponding to the null hypothesis of no association. (DOC) [file pone.0080642.s002.doc]

**
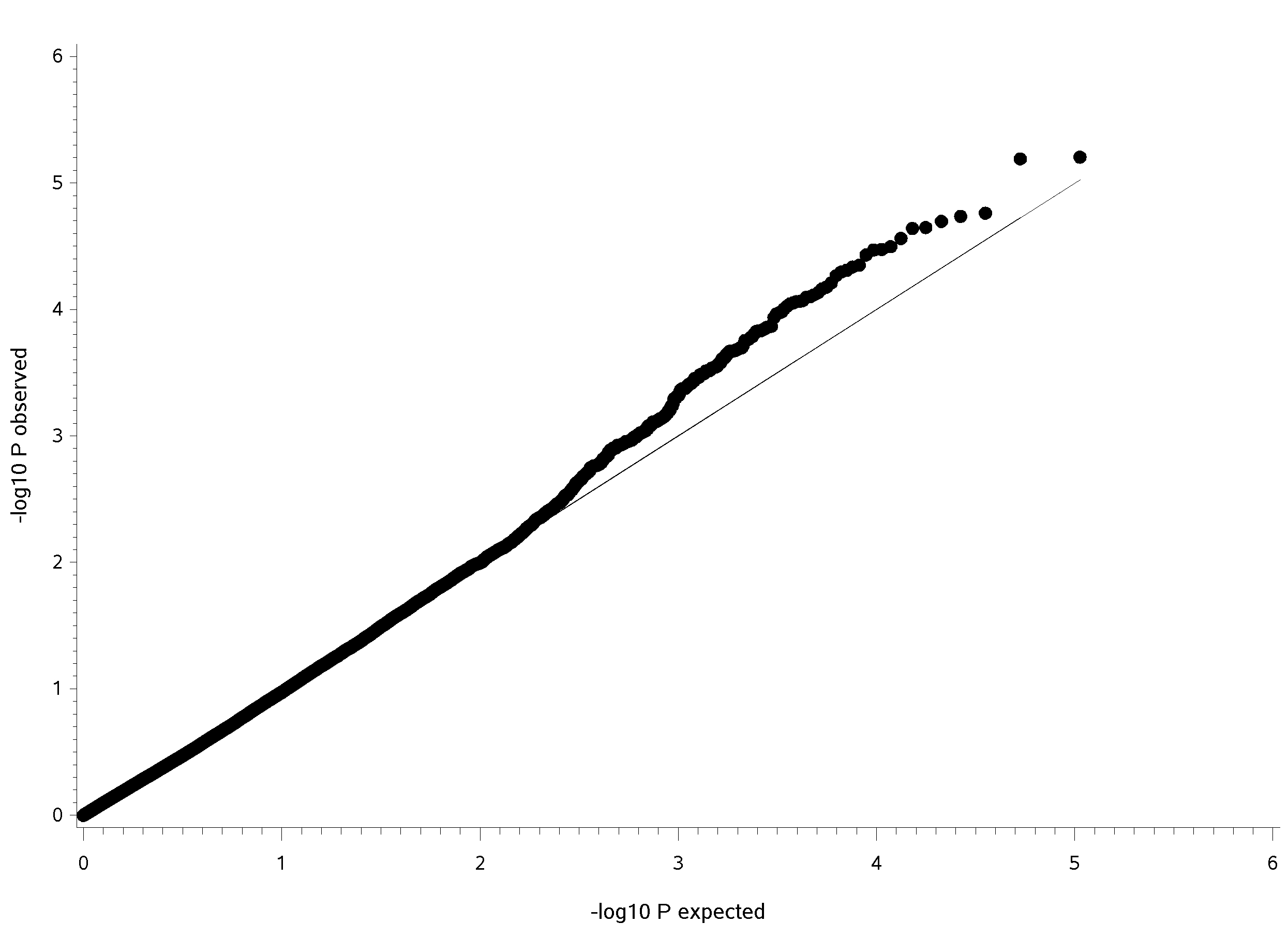
**

**Figure S2.** **Q-Q-plot of expected –log_10_P-values versus observed–log_10_P-values from the mixed linear model analysis for congenital sensorineural deafness in Dalmatian dogs with bilateral deafness.** Shown are all 106,435 SNPs included in the genome-wide association analysis with the grey line corresponding to the null hypothesis of no association.
