# Supplementary material for: Congenital Sensorineural Deafness in Dalmatian Dogs Associated with Quantitative Trait Loci
Source: PLoS One. 2013 Dec 4;8(12):e80642. doi: 10.1371/journal.pone.0080642 (PMC3851758; doi:10.1371/journal.pone.0080642)
Supplement: Table S1 — Summary of results for the genome-wide association study employing a mixed linear model for canine congenital sensorineural deafness in Dalmatian dogs using all deaf dogs as cases and all hearing dogs as controls. The SNP-ID, the position on dog chromosome (CFA) in base pairs (bp) according to CanFam3.1 and CanFam2.0, minor allele, minor allele frequency (MAF) for all, affected (MAFa) and unaffected (MAFu) dogs (controls), variance explained by the respective SNP (VE) and -log10P-values (-log10P) from the mixed linear model analysis are given. Odds ratios (OR) are from a case-control study stratified by sex with 95% confidence intervals (CI). (DOC) [file pone.0080642.s004.doc]

**Table S1.** **Summary of results for the genome-wide association study employing a mixed linear model for canine congenital sensorineural deafness in Dalmatian dogs using all deaf dogs as cases and all hearing dogs as controls.** The SNP-ID, the position on dog chromosome (CFA) in base pairs (bp) according to CanFam3.1 and CanFam2.0, minor allele, minor allele frequency (MAF) for all, affected (MAF_a_) and unaffected (MAF_u_) dogs (controls), variance explained by the respective SNP (VE) and -log_10_P-values (-log_10_P) from the mixed linear model analysis are given. Odds ratios (OR) are from a case-control study stratified by sex with 95% confidence intervals (CI).

| CFA | Position | Position | SNP-ID | Minor | MAF | MAF_a_ | MAF_u_ | VE | OR | CI-L | CI-U | -log_10_P |
| --- | --- | --- | --- | --- | --- | --- | --- | --- | --- | --- | --- | --- |
|  | CanFam3.1 | CanFam2.0 |  | allele |  |  |  |  |  |  |  |  |
| 2 | 13,786,700 | 16,675,682 | BICF2P176848 | T | 0.08 | 0.17 | 0.03 | 0.13 | 7.02 | 3.22 | 15.36 | 6.72 |
| 6 | 45,474,835 | 48,537,551 | TIGRP2P83893_ | A | 0.49 | 0.66 | 0.42 | 0.09 | 2.70 | 1.77 | 4.12 | 4.11 |
|  |  |  | RS8732055 |  |  |  |  |  |  |  |  |  |
| 6 | 68,927,940 | 71,986,567 | BICF2P590845 | A | 0.14 | 0.17 | 0.13 | 0.09 | 1.35 | 0.78 | 2.33 | 5.58 |
| 14 | 39,561,348 | 42,518,712 | BICF2G630529431 | T | 0.17 | 0.16 | 0.18 | 0.07 | 1.12 | 0.67 | 1.89 | 5.57 |
| 27 | 9,400,352 | 12,412,335 | BICF2S23410492 | C | 0.05 | 0.13 | 0.02 | 0.09 | 9.09 | 3.34 | 24.6 | 5.38 |
| 29 | 23,903,462 | 26,901,967 | BICF2G630625485 | T | 0.20 | 0.21 | 0.20 | 0.09 | 1.06 | 0.67 | 1.71 | 6.08 |
| 17 | 28,929,911 | 32,032,913 | BICF2G630212376 | A | 0.11 | 0.18 | 0.08 | 0.09 | 2.63 | 1.45 | 4.72 | 5.85 |
| 18 | 51,795,260 | 54,820,227 | BICF2P28982 | G | 0.16 | 0.17 | 0.15 | 0.07 | 1.19 | 0.71 | 1.99 | 4.01 |
| 27 | 25,549,421 | 28,568,843 | BICF2P507470 | T | 0.11 | 0.21 | 0.06 | 0.03 | 3.88 | 2.14 | 7.02 | 4.81 |
| 31 | 30,836,962 | 32,955,387 | BICF2G630740465 | G | 0.20 | 0.29 | 0.16 | 0.11 | 2.19 | 1.38 | 3.47 | 5.57 |
