# Supplementary material for: Congenital Sensorineural Deafness in Dalmatian Dogs Associated with Quantitative Trait Loci
Source: PLoS One. 2013 Dec 4;8(12):e80642. doi: 10.1371/journal.pone.0080642 (PMC3851758; doi:10.1371/journal.pone.0080642)
Supplement: Table S2 — Chromosomal loci identified as significantly associated with canine congenital sensorineural deafness, compared to a control group of 157 hearing Dalmatian dogs. (DOCX) [file pone.0080642.s005.docx]

**Table S2. Chromosomal loci identified as significantly associated with canine congenital sensorineural deafness, compared to a control group of 157 hearing Dalmatians dogs.**

| Group of Dalmatian dogs | N | Chromosome (CFA) locations of CCSD-associated loci | | | | | | | |
| --- | --- | --- | --- | --- | --- | --- | --- | --- | --- |
| All deaf Dalmatian dogs | 78 | 2 | 6/6^*^ | 14 | 17 | /18^*^ | 27/27^*^ | 29 | 31 |
| Deaf with brown eyes | 61 | 2 | 6 (2 loci)^§^ | /14^*^ |  |  | 27 | 29 |  |
| Deaf with blue eyes | 17 |  |  |  | 17 | 18 | 27 |  | 31 |

/ ^*^ = loci identified on secondary analyses.

^§^ Two loci were identified on chromosome CFA6.
