# Supplementary material for: Congenital Sensorineural Deafness in Dalmatian Dogs Associated with Quantitative Trait Loci
Source: PLoS One. 2013 Dec 4;8(12):e80642. doi: 10.1371/journal.pone.0080642 (PMC3851758; doi:10.1371/journal.pone.0080642)
Supplement: Table S3 — Distribution (%) of canine congenital sensorineural deafness (CCSD) in Dalmatian dogs by the genotypes of significantly associated SNPs. With exception of SNP BICF2P176848, only each one genotype per SNP is highly associated with CCSD-affection. (DOC) [file pone.0080642.s006.doc]

**Table S3.** **Distribution (%) of canine congenital sensorineural deafness (CCSD) in Dalmatian dogs by the genotypes of significantly associated SNPs.** With exception of SNP BICF2P176848, only each one genotype per SNP is highly associated with CCSD-affection.

| SNP-ID | SNP-allele | | Genotypes | | |
| --- | --- | --- | --- | --- | --- |
|  | 1 | 2 | 1/1 | 1/2 | 2/2 |
| Brown eye color | | | | | |
| BICF2P176848 | C | T | 21.4 | 67.9 | 100.0 |
| TIGRP2P83893_  RS8732055 | A | G | 61.4 | 20.4 | 12.8 |
| BICF2P590845 | A | G | 80.0 | 22.5 | 27.6 |
| BICF2G630529431 | C | T | 31.5 | 17.9 | 100.0 |
| BICF2S23410492 | C | T | 0 | 78.3 | 22.1 |
| BICF2G630625485 | G | T | 28.0 | 24.7 | 80.0 |
| Blue eye color | | | | | |
| BICF2G630212376 | A | G | 100.0 | 4.0 | 8.9 |
| BICF2P28982 | A | G | 100.0 | 7.8 | 9.1 |
| BICF2P507470 | G | T | 6.1 | 24.0 | 100.0 |
| BICF2G630740465 | A | G | 6.1 | 7.7 | 83.3 |
