# Supplementary material for: Congenital Sensorineural Deafness in Dalmatian Dogs Associated with Quantitative Trait Loci
Source: PLoS One. 2013 Dec 4;8(12):e80642. doi: 10.1371/journal.pone.0080642 (PMC3851758; doi:10.1371/journal.pone.0080642)
Supplement: Table S4 — Distribution (%) of the genotypes significantly associated with canine congenital sensorineural deafness (CCSD) for controls and CCSD-affected Dalmatian dogs. The genotypes conferring high risk to CCSD are given in bold. (DOC) [file pone.0080642.s007.doc]

**Table S4.** **Distribution (%) of the genotypes significantly associated with canine congenital sensorineural deafness (CCSD) for controls and CCSD-affected Dalmatian dogs.** The genotypes conferring high risk to CCSD are given in bold.

| SNP-ID | SNP-allele | | Genotypes | | | | | |
| --- | --- | --- | --- | --- | --- | --- | --- | --- |
|  | 1 | 2 | 1/1 | 1/2 | 2/2 | 1/1 | 1/2 | 2/2 |
|  |  |  | Controls |  |  | CCSD-affected | | |
| Brown eye color |  |  |  |  |  |  |  |  |
| BICF2P176848 | C | T | 0.94 | 0.06 | 0.0 | 0.66 | **0.31** | **0.03** |
| TIGRP2P83893_  RS8732055 | A | G | 0.12 | 0.60 | 0.28 | **0.49** | 0.40 | 0.11 |
| BICF2P590845 | A | G | 0.01 | 0.24 | 0.75 | **0.07** | 0.18 | 0.75 |
| BICF2G630529431 | C | T | 0.65 | 0.35 | 0.0 | 0.77 | 0.12 | **0.12** |
| BICF2S23410492 | C | T | 0.0 | 0.03 | 0.97 | 0.0 | **0.30** | 0.70 |
| BICF2G630625485 | G | T | 0.61 | 0.39 | 0.01 | 0.60 | 0.33 | **0.07** |
| Blue eye colour |  |  |  |  |  |  |  |  |
| BICF2G630212376 | A | G | 0.0 | 0.15 | 0.85 | **0.13** | 0.06 | 0.81 |
| BICF2P28982 | C | T | 0.0 | 0.30 | 0.70 | **0.12** | 0.24 | 0.65 |
| BICF2P507470 | G | T | 0.87 | 0.13 | 0.0 | 0.41 | 0.47 | **0.12** |
| BICF2G630740465 | A | G | 0.69 | 0.31 | 0.01 | 0.44 | 0.25 | **0.31** |
